# Is justice served by reliance on ICD and DSM classifications of mental disorder in medico-legal reporting?

**DOI:** 10.1192/bja.2025.10142

**Published:** 2026-01

**Authors:** Keith Rix

**Affiliations:** a Door Tenant, The Chambers of Mark Love, Inner Temple, London, UK; Visiting Professor of Medical Jurisprudence at the Faculty of Medicine, Dentistry and Life Sciences, University of Chester, Chester, UK; and an Honorary Associate Professor at Norwich Medical School, University of East Anglia, Norwich, UK. He is a retired forensic psychiatrist and a co-editor of *Rix’s Expert Psychiatric Evidence* (Cambridge University Press). He has been awarded honorary fellowships of the Faculty of Forensic and Legal Medicine of the Royal College of Physicians and the Expert Witness Institute.

**Keywords:** DSM, expert evidence, ICD, justice, medico-legal

## Abstract

The histories of the DSM and ICD classifications are set out so as to identify weaknesses and limitations that can affect their application in medico-legal reporting. These are illustrated by reference to published judgments and three detailed case studies. The analysis and case studies identify how expert witnesses’ reliance on the DSM and ICD can be challenged in order to seek to undermine their evidence.

## LEARNING OBJECTIVES

After reading this article you will be able to:

- understand the nature and purposes of the ICD and DSM classifications
- understand how the ICD and DSM have been and are used in legal proceedings
- understand the limitations of the ICD and/or DSM in medico-legal reporting and how they can be exposed in the testing of expert psychiatric evidence.

‘[T]he majority of patients do not conform to the tidy stereotyped descriptions found in textbooks. They possess some, but not all, of the symptoms of two or three different diagnostic categories and so have to be allocated more or less arbitrarily to whichever syndrome they most nearly resemble. As a result disagreements about diagnosis are frequent’

(Kendell 1975: p. 3)

‘All our diagnostic terms are simply concepts and all our definitions more or less arbitrary and […] there is nothing God-given or immutable about the categories and definitions of their official nosology’

(Kendell 1991)

When Professor Michael Kopelman referred to the *International Classification of Diseases* (ICD) and the *Diagnostic and Statistical Manual of Mental Disorders* (DSM) as ‘those bloody books’ (*United States of America v Assange* [2021]), he was probably speaking for many psychiatrists and lawyers. In the words of one of this article’s reviewers, ‘ the use of ICD/DSM in legal proceedings has evolved from seldom (1980s) to optional extras (roughly 1990–2005) to almost obligatory (approximately 2005 – present), a big change in 40 years!’ The purpose of this article is to explain what the ICD and the DSM are, describe their use in legal jurisdictions in the British Isles and identify limitations that may be exposed in expert psychiatric evidence when reliance on them is tested. In a companion article in this issue (Rix 2025) I discuss misperceptions, misunderstandings and misuse of ICD and DSM classifications in medico-legal reporting.

## What the ICD and DSM are (and are not)

### The nature and purposes of the ICD and DSM

Comparing DSM and ICD, Tyrer (2014) refers to how ‘[w]ithout a classification system the necessary economical communication with colleagues to convey information becomes a lengthy description of clinical problems that is self-defeating’. Both are used for communication and have some similarities, but there are significant limitations, and also differences, that affect their utility in medico-legal reporting.

The differences partly reflect the different priorities of the World Health Organization (WHO) and the American Psychiatric Association (APA). The WHO’s ICD is a comprehensive classification of mental, behavioural and neurotypical disorders for use by a wide range of health professionals in countries of very varied sizes, cultures and resources, whereas the APA’s DSM is designed to meet the needs of one, or perhaps two, professions – psychiatrists and psychologists – in a single country (Kendell 1991).

Clark et al (2017) note that they share ‘many features (e.g., a categorical structure) that respond to the purposes for which they were developed and primarily used, which include compilation of health statistics, allocation of mental-health resources, clinical communication, and decision making in regulatory, legal, and health-insurance systems, all ultimately in service of public mental-health-care needs’. Whether developed for use in legal systems is questionable but they are used.

The limitations of the ICD and DSM are that although both are regarded as categorical systems, this is questionable. Mental disorders are non-taxonomic. They are not unique, discrete entities. Most exist on dimensions. There are arbitrary boundaries on the ‘normality–pathology’ dimension, requiring a ‘threshold boundary’, and on the dimensions connecting overlapping disorders, between which there is no ‘zone of rarity’ and which therefore require a ‘disorder boundary’.

Using the example of depression, Tyrer (2014) illustrates the requirement for a threshold boundary, but notes that the threshold is not a clean one; many people just below threshold have depressive symptoms that do not qualify for a diagnosis even though research shows that they may be as unwell as others just over the threshold. Using the example of anaemia, Tyrer illustrates graphically the zone of rarity between iron-deficiency anaemia and pernicious anaemia. They do not overlap. As Kendell (1991) says, about psychiatric syndromes, it has not been possible to identify points of rarity that separate individual syndromes from their neighbours.

**FIG 1:**
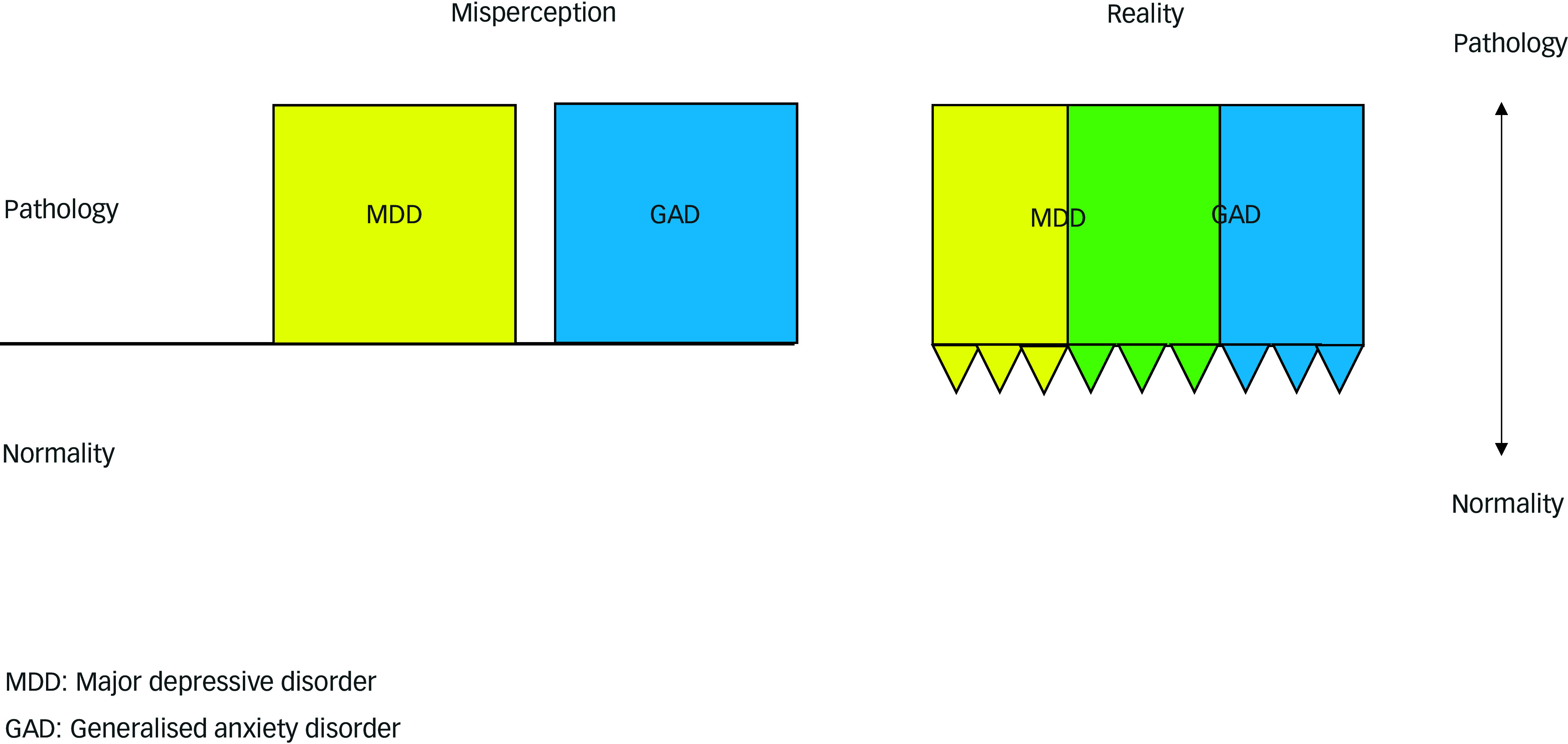
How misapplication of DSM categorisation can mislead as to the nature of major depressive disorder and generalised anxiety disorder.

There is no zone of rarity between DSM’s major depressive disorder and generalised anxiety disorder (Zhou 2017). Figure 1 illustrates how DSM categorisation, when applied rigidly and without the required flexibility and clinical judgement, misleads as to the nature of major depressive disorder and generalised anxiety disorder. As the introduction to ICD-11 (WHO 2024) states, both of these boundaries represent key issues in diagnosis, so unsurprisingly they also represent key issues when diagnoses are used in medico-legal reporting. Experts who rely on these boundaries to diagnose, or not diagnose, mental disorders or to distinguish one from another risk misleading the court.

ICD and DSM are better regarded as nomenclatures than classifications.

### The history of the ICD and DSM

In 1948, the WHO agreed to use its International Statistical Classification of Diseases, Injuries and Causes of Death, the first version of which originated in the first International Statistical Congress in Brussels in 1853, as ‘a global common language for defining and communicating about diseases and health conditions’ (Clark 2017), thus supporting the WHO’s objective of achieving the highest level of health for all people by pursuing universal healthcare. This public health focus was fundamental to ICD-6 (1949) and it has remained so. In ICD-8 (1966) the WHO made clear that the purpose of its glossary was to facilitate diagnosis in clinical settings as well as serve as a statistical classification system. When ICD-10 was issued in 1992, it was described as being ‘intended for general clinical, educational and service use’ (WHO 1992: p. 1).

The DSM also has its roots in the 19th century. It grew out of the statistical manuals used to document diagnoses of patients in US psychiatric institutions. However, both classifications can be traced back to Brigadier General William Menninger, MD, who was Chief of the US Army Medical Corps’ Psychiatric Division during the Second World War and developed a mental disorder classification better suited to military medicine. After the war he recognised a need for a diagnostic manual suitable for civilian practice and developed, for use by the Veterans Health Administration, the Nomenclature of Psychiatric Disorders and Reactions (Office of the Surgeon General 1946). In 1948, the WHO drew heavily on this when it published ICD-6 and the APA did likewise when it published its first DSM in 1952 (DSM-I).

There is a misconception that the ICD is not used in the USA. Adapted ICD codes, known as ‘Clinical Modifications’, are required for Medicare billing and reimbursement, for coding morbidity and healthcare utilisation from patient records, and to provide statistical returns to the US National Center for Health Statistics.

### The processes of revision

For more than 60 years countless committees have produced updated and revised versions of the ICD and DSM. Thus, they are based on consensus about clusters of clinical symptoms. They are not based on any objective laboratory measure or biological marker or on research that has established that a particular disorder can be sufficiently distinguished from other similar disorders, in terms of its epidemiology, response to treatment and prognosis, to justify its recognition as a discrete entity. Explaining why the National Institute of Mental Health (NIMH) would no longer fund research based on DSM-5, Thomas R. Insel, Director of NIMH, said:

‘The weakness is its lack of validity. Unlike our definitions of ischemic heart disease, lymphoma, or AIDS, the DSM diagnoses are based on a consensus about clusters of clinical symptoms, not any objective laboratory measure’ (Insel 2013).

Where expert witnesses rely on DSM, or ICD, diagnoses in court, they can expect to be challenged as to their lack of validity and the absence of research that supports their delineation.

In the case of ICD, there are committees of representatives from nearly 200 WHO member states, so the ICD represents international consensus as to the detailed descriptions of conditions that are used for a process mainly of prototypical diagnosis, matching a person’s psychopathology to a detailed description in the guidelines. The description requires a face validity acceptable in as many member states as possible. The classification has to accommodate cultural variations in psychopathology, clinical judgement and diagnostic practice (First 2021). Global applicability is paramount. The prioritisation of public health needs, based on the priorities of sponsoring organisations, has been proposed as the reason why ICD-11 includes 11 disorders that are not in DSM-5 (Clark 2017).

DSM criteria are used for an algorithmic approach to diagnosis, where criteria are counted (‘≥5 symptoms’), time periods specified (‘during the same 2-week period’) and exclusion criteria specified. The basis for the algorithmic approach is questionable. Paris (2015: p. 9) has observed: ‘But if a typical DSM diagnosis requires, for example, five out of nine criteria, nobody knows whether four or six criteria would have been more or less valid’.

The over-rigid application of the DSM and ICD criteria is illustrated by the case of *R (B) v Dr SS, Responsible Medical Officer* [2005] (Box 1).

BOX 1 An over-rigid application of the DSM and ICD criteria

The issue was whether Mr B, detained under the Mental Health Act 1983 might lawfully be given treatment without his consent. Professor H, instructed as an independent expert by his solicitors, argued that because Mr B did not meet, and had not met, the diagnostic criteria for hypomania or mania set by DSM-IV (and ICD-10) he should not be treated compulsorily. The criteria set minimum duration requirements of 4 days for hypomania and 7 days for mania.

The court accepted submissions that the DSM (and ICD) guidelines were just that, 4 days was an arbitrary minimum and it was a fair criticism of Professor H’s reports and evidence that he adopted an over-rigid application of the DSM and ICD criteria.

It concluded that the questions that fall to be considered and answered in law should not be dictated by any rigid application of diagnostic criteria.

(*R (on the application of B) v Dr SS, Responsible Medical Officer* [2005])

Agreement as to diagnosis can conceal significant disagreement, which can be difficult to explain in court, especially if the court wants to believe that these diagnostic systems are absolute and unshakeable:

‘It is also possible for two examiners to converge on the same diagnosis without any overlap in the specific underlying criteria. In this scenario, the examiners have 100% inter-rater reliability with respect to the diagnosis but 0% validity in terms of the underlying signs and symptoms related to their conclusions’ (Hagan 2015).

When the nature of a person’s mental condition is at issue, the court is likely to need to know how the diagnosis is made and about the validity of the diagnostic category. This may require explanations about the roles of committees, political influences, pressure groups and the financial interests of pharmaceutical companies (McHugh 1999; Paris 2015; Kapp 2020; Horwitz 2021). There may be questions about the scientific basis for distinctions based on algorithmic criteria.

The limitations of the ICD and DSM diagnostic categories are illustrated in general clinical practice, where there are high rates of use of the categories ‘not otherwise specified’ (ICD) and ‘not elsewhere defined’ (DSM) (Clark 2017). These are the categories into which the baby gets thrown with the bathwater. In the everyday practice of the real world, where, to quote Kendell (1975: p. 3), ‘the majority of patients do not conform to the tidy stereotyped descriptions found in textbooks’, there will be more babies thrown out with the bathwater than are left in the bath or, in the language of Clark et al (2017), more in the ‘wastebasket categories’. The value of a diagnostic system that works in only a minority of patients is questionable. It calls into question how typical, for example, the defendant with ICD or DSM schizophrenia is of all people with schizophrenia.

*D v The Bishop’s Conference of Scotland* [2022] (Box 2) is a case in which the court found psychiatric injury even though D’s condition did not fall within DSM-5 (American Psychiatric Association 2013).

BOX 2 When the pursuer’s condition did not conform to any pathological entity described in DSM-5

As a teenager, D attended a residential school in Scotland where pupils were aiming to become priests in the Catholic Church. D was sexually abused there by a priest. D became a priest for a lengthy period but eventually left. He claimed damages for loss said to have been caused by the abuse, including having to leave the post.

In relation to whether the pursuer suffered a psychiatric injury, the court said that the first question was whether the alleged injury was required to fall within DSM-5 or ICD-11 and referred to *Rorrison* v *West Lothian College* [1999] (see Box 5), where the court explained that reference to these classifications was helpful as a matter of fair notice, but what constitutes a recognised disorder is a matter for expert evidence, so those systems were not necessarily conclusive.

Dr L, the expert instructed for the pursuer, did not state in terms that he had suffered any form of diagnosed psychiatric injury other than panic disorder, nor did Professor M, the expert instructed for the defender. However, Dr L was using DSM-5 as the sole basis for deciding whether a defined or classified psychiatric injury existed. When his evidence was considered in the round, there was other evidence from him which pointed clearly towards psychiatric injury at that time, albeit not falling within DSM-5, and the court concluded that D sustained psychiatric injury caused by the abuse.

(*D v The Bishop’s Conference of Scotland* [2022])

Autism spectrum disorder illustrates the difference between the algorithmic and prototypical approaches (First 2021). For persistent deficits in social communication/social interaction, DSM-5-TR (American Psychiatric Association 2022) requires all three specific manifestations, whereas ICD-11 (WHO 2024) states that the ‘manifestations *may* include the following’ (emphasis added) and lists seven manifestations, three of which correspond to the DSM-5 requirements. For restrictive, repetitive patterns of behaviour, ICD-11 provides a list of seven items as examples, but DSM-5 is more prescriptive as it requires two out of a list of four items.

These are matters that need to be taken into consideration when deciding what weight to attach to ICD or DSM diagnoses and to the absence of a diagnosis where criteria are not met.

### Proceed with caution

There is some recognition of these limitations in the introductions to both the ICD (Box 3) and DSM (Box 4).

BOX 3 Statements as to the use of the ICD

‘These descriptions and guidelines carry no theoretical implications, and they do not pretend to be comprehensive statements about the current knowledge of the disorders. They are simply a set of symptoms and comments that have been agreed, by a large number of advisors and consultants in many different countries, to be a reasonable basis for defining the limits of categories in the classification of mental disorders.’

‘Statements about the duration of symptoms are also intended as general guidelines rather than strict requirements; clinicians should use their own judgement about the appropriateness of choosing diagnoses when the duration of particular symptoms is slightly longer or shorter than that specified.’

(ICD-10: World Health Organization, 1992: p. 2)

‘The [Clinical Descriptions and Diagnostic Requirements] (CDDR) are written to allow for the exercise of clinical judgement, and it is the diagnosing health professional who is responsible for developing a diagnostic formulation appropriate for an individual patient, considering the patient’s individual, social and cultural context as well as the characteristics of the health system. It is equally important to note that diagnostic classification is only a part of patient assessment. The CDDR are not a guide to patient care, nor a comprehensive textbook of psychiatry, nor a manual of how to conduct clinical assessments and differential diagnoses. The focus of the CDDR is on the classification of disorders and not the assessment and treatment of people, who are frequently characterized by multiple disorders and diverse needs.’

(ICD-11: World Health Organization, 2024: p. 3)

These cautionary statements make clear that the use of the ICD and DSM is to be informed by clinical judgement and they call for flexibility. These publications are neither comprehensive statements about the current knowledge of the disorders nor comprehensive textbooks of psychiatry.

Given these cautionary notes it seems somewhat surprising that a psychiatric expert in *Ludlow v National Power Plc* [2000] should have been critical of the DSM as being too formulaic to be discriminating and to have said that it marginalised clinical judgement and common sense.

Few courts and tribunals seem to be aware, or advised by experts, of such warnings, notwithstanding the fact that in DSM-5 they appear under a bold heading ‘Cautionary Statement for Forensic Use of DSM-5’. In *McTear v Imperial Tobacco Ltd* [2005] an expert agreed that the passage in DSM-IV (Box 4) was, in general terms, the correct approach and said that it urged the use of caution in the drawing of conclusions from the fact that an individual’s presentation met the criteria for a DSM-IV diagnosis.

BOX 4 Statements as to the use of the DSM

‘Although this manual provides a classification of mental disorders, no definition adequately specifies precise boundaries for the concept “mental disorder” […] There is no assumption that each mental disorder is a discrete entity with sharp boundaries (discontinuity) between it and other mental disorders.’

(DSM-III: American Psychiatric Association, 1980: p. xxii)

‘In most situations, the clinical diagnosis of a DSM-IV mental disorder is not sufficient to establish the existence for legal purposes of a “mental disorder,” “mental disability,” “mental disease,” or “mental defect.”’

‘The diagnostic categories, criteria and textual descriptions are meant to be employed by individuals with appropriate clinical training and experience in diagnosis. … The specific diagnostic criteria … are meant to serve as guidelines to be informed by clinical judgement and not meant to be used in a cookbook fashion.’

(DSM-IV: American Psychiatric Association, 1994): p. xxiii)

‘DSM-5 is intended to serve as a practical, functional, and flexible guide for organizing information […] Diagnostic criteria are offered as guidelines for making diagnoses, and their use should be informed by clinical judgment […]’

‘However, the use of DSM-5 should be informed by an awareness of the risks and limitations of its use in forensic settings. When DSM-5 categories, criteria and textual descriptions are employed for forensic purposes, there is a risk that diagnostic information will be misused or misunderstood. These dangers arise because of the imperfect fit between the questions of ultimate concern to the law and the information contained in a clinical diagnosis. In most situations, the clinical diagnosis of a DSM-5 disorder […] does not imply that an individual with such a condition meets legal criteria for the presence of a mental disorder or a specific legal standard […] As a result, it is important to note that the definition of mental disorder included in DSM-5 was developed to meet the needs of clinicians, public health professionals, and research investigators rather than all of the technical needs of the courts and legal professionals.’

(DSM-5: American Psychiatric Association, 2013: p. 25)

Having regard to these warnings, any reliance on the ICD or DSM in making diagnoses in evidence for courts and tribunals has to be viewed with caution.

Such caution is also necessary where there has been authoritative criticism of the ICD or DSM, such as the criticism by Kopelman & Fleminger (2002) of the organisation and definitions of neuropsychiatric conditions in DSM-IV and ICD-10, many of which criticisms still hold, and Kopelman’s (2022) criticism of ICD-11’s definitions of Korsakoff syndrome and alcohol-related brain damage.

## The use of the DSM and ICD in courts and tribunals

### Personal injury litigation

*Rorrison v West Lothian College* [1999] (Box 5) illustrates how it is common practice in personal injury pleadings in Scotland to aver that a pursuer was or is suffering from a specified condition recognised in the DSM or ICD. When a similar issue arose in *Laudanska v University Abertay* [2003] the court referred to the ‘hypothesis’ that the decision in *Rorrison* was correct and how it was not convinced that it should be automatically followed; but it was. In *Mather v British Telecommunications Plc* [2000], the pursuer averred that she had developed and suffered from a panic disorder with agoraphobia in DSM-IV criteria. The court found that she had plainly averred that she had suffered from a recognised psychiatric illness.

BOX 5 Failure to refer to the DSM or ICD

On 17 August 1992 Angela Rorrison started work at West Lothian College as a welfare auxiliary in charge of the first aid room and its cabinet. In late December 1992 she was marched to the first aid room by the personnel officer, the safety officer and the trade union health and safety representative. She felt humiliated as she walked past other staff. She was criticised and humiliated for keeping asthma inhalers in the cabinet, something that had previously been authorised by the college registrar. The safety officer stood by the door. The health and safety representative shouted and paced. The personnel officer repeated the health and safety representative’s words in a patronising manner. She felt trapped, threatened and embarrassed. She was ordered to clear out the cabinet. She was upset, had a severe headache and was unable to sleep.

There were further similar incidents. It was Ms Rorrison’s case that, as a result of the personnel officer’s treatment of her, during the latter part of 1993 she began to experience further psychological distress: palpitations, sweating, over-breathing and feelings of panic. She was prescribed a beta-blocker.

In relation to damages it was averred: ‘She suffered psychological damage in the form of severe anxiety and depression. She has not worked since suffering a nervous breakdown on 29 March 1994 … She was examined by a clinical psychologist who diagnosed her as having “understandable and justifiable psychological distress”. … She suffered severe anxiety, panic attacks and loss of confidence and self-esteem. She had acute psychological distress. She suffered depression. She continues to become visibly distressed and tearful when thinking about her experiences with the defenders’.

The court observed that in practice, it was common for pleadings to aver that a pursuer was diagnosed by a psychiatrist as suffering from a specified condition, or to aver more shortly that the pursuer was suffering from a specified condition in DSM-IV or in ICD-10. In the present case, no disorder recognised in DSM-IV was pleaded; and there was no suggestion that the position was any different in relation to ICD-10. The case was dismissed because, as there was no suggestion that Ms Rorrison had ever been diagnosed as suffering from a recognised psychiatric disorder and there was no suggestion that her condition was recognised by any psychiatrist or body of psychiatric opinion as constituting a psychiatric disorder, her action based on negligence could not succeed.

(*Rorrison v West Lothian College* [1999])

In *Hussain v The Chief Constable of West Mercia Constabulary* [2008], the court said: ‘A recognised psychiatric illness is one which has been recognised by the psychiatric profession. In general, they are illnesses that are within the ICD […]’.

Although the Judicial College *Guidelines for the Assessment of General Damages in Personal Injury Cases* (Judicial College 2024) does not advise the use of the ICD or DSM, its guidance on awards for post-traumatic stress disorder refers to how it relies on ‘cases which variously reflect the criteria established’ (p. 14) in DSM-IV and DSM-5.

### Professional regulation

The General Medical Council and the Nursing and Midwifery Council use the ICD when referring to psychiatric diagnoses (e.g. *Stefan v General Medical Council (Health Committee of the General Medical Council)* [2001]).

### Employment litigation

The Employment Tribunal, which is concerned with ‘mental impairment’ resulting from, or consisting of, a mental illness only if the illness is ‘clinically well recognised’, applies guidance issued by the Department for Education and Employment which states that it is very likely that a respected body of medical opinion would include as clinically well-recognised those illnesses specifically mentioned in publications such as the ICD. However, the use of ‘include’ and ‘such as’ does allow for reliance on diagnoses that are not in the ICD. This was recognised in *Morgan v The Staffordshire University* [2002], where, in addition to proof of a mental illness specifically mentioned in the ICD, the tribunal recognised at least two other routes to establishing ‘mental impairment’: (a) proof of a mental illness specifically mentioned in a publication, ‘such as’ the ICD, of very wide professional acceptance; (b) proof by other means of a medical illness recognised by a respected body of medical opinion.

Failure by a psychiatrist to make what the court called ‘a statement to the effect of whether the recurrent depressive illness comprised (in his view) a recurrent depressive disorder (or some other illness) for the purposes of ICD-10’ in *Woods v Royal College of Nursing* [2003] resulted in the applicant’s failure to succeed on the issue of disability. The tribunal did state that it might have been that, if the applicant had produced an expert’s report guiding the tribunal as to the type of illness from which she suffered (and whether it was contained within the ICD and if so where), the tribunal would have made a different decision. Likewise in *Wilson v Southern Counties Fuels Ltd* [2004], the tribunal referred to the failure to comply with guidelines as a bad start. This was a case in which there was a diagnosis of ‘clinical depression’ but the tribunal said that what was required was ‘to bring someone who is, or has been, suffering from depression within one of the clinically well recognised illnesses’. The term ‘clinical depression’ is for many psychiatrists synonymous with a depressive illness or episode with biological or somatic symptoms, and very much a clinically well-recognised illness. The outcome of both of these cases might have been different if reference had been made to ICD-10 or, in the case of *Wilson*, a statement that a depressive illness or episode with biological or somatic symptoms is clinically well-recognised.

### Immigration and asylum cases

Another example of a court or tribunal regarding it as a failure not to use the DSM or ICD is in the Asylum and Immigration Tribunal (AIT). In 2005 a judge in an AIT referred to failure to apply DSM-IV or ICD-10 as indicative of a lack of rigour on the part of the expert (*DE (suicide, psychiatric treatment, J applied) Turkey* [2005]).

### Health, Education and Social Care Chamber (Mental Health)

The Tribunals Judiciary (2019) Practice Direction on statements and reports in mental health cases before the First-tier Tribunal (Mental Health) (Health, Education and Social Care Chamber) states that the report must include ‘whether the patient is now suffering from a mental disorder and, if so, whether a diagnosis has been made, what the diagnosis is, and why. There is no requirement to use any particular classification or nomenclature’ (para. 12(f)).

A comment in the Upper Tribunal (Mental Health) (Health, Education and Social Care Chamber) gives an indication of the position that might be taken when the issue is one of classification for the purposes of the Mental Health Act 1983. In *DL-H v Devon Partnership NHS Trust v Secretary of State for Justice* [2010], the tribunal made this comment for any value it might have in the future, pending consideration by the Upper Tribunal in an appropriate case:

‘The answer cannot depend on the manual that happens to be used […] There must be an answer that provides protection for patients from vague or differing definitions while ensuring that those who present a danger are not left free to harm themselves or others for failing to meet over-prescriptive criteria’.

The case of *B* outlined in Box 1 illustrates what happens when the diagnostic criteria are used ‘over-prescriptively’.

It is doubtful that if the Mental Health Bill 2025 (for England and Wales) is enacted without changes to Clause 1(3), the position will be any different. This clause includes a reference to ‘psychiatric disorder’, which ‘means mental disorder other than autism or learning disability’ (the latter being a term which it is reasonable to regard as applying to ICD-11’s ‘disorders of intellectual ability’ and DSM-5’s ‘intellectual disability (intellectual development disorder)’. It does not appear that this new form of categorisation of mental disorders will affect any reliance on the ICD or DSM.

Provided that, if there is reliance on the DSM for a diagnosis of autism, the requirement to satisfy all three criteria for persistent deficits in social communication and social interaction or at least two out of four manifestations of restricted repetitive patterns of behaviour, interests or activities, is not interpreted over-prescriptively, there should be no problem. It is doubtful that the tribunal will decide that someone does not have autism on the basis that only two of the three social communication criteria are satisfied or just one manifestation of restrictive, repetitive patterns of behaviour, interests or activities.

There are minor definitional differences between ICD-11’s ‘disorders of intellectual disability’ and DSM-5’s ‘intellectual disability (intellectual development disorder)’. However, as both include what amounts to significant impairment of intelligence, which is the only specified feature of ‘learning disability’ in Clause 1(3), these minor definitional differences should be of no consequence.

### Social security cases

In a social security case in England and Wales, *DE v Secretary of State for Work and Pensions (PIP) (Personal independence payment)* [2021], it was held that a tribunal ‘should refer to the DSM-5 if considering a case involving Alcohol Use Disorder’. The Social Security Appeals Tribunal Northern Ireland does not regard reference to the ICD as essential. However, in (Unnamed) [2003], an unsuccessful appeal to the Social Security and Child Support Commissioner, it was said that it would have helped. The claimant had relied on a letter from a general practitioner and it was noted by the tribunal that, although it referred to the claimant being depressed, there was no ‘clinical diagnosis’ made by the general practitioner ‘in accordance with’ the ICD.

### Police pension cases

It is common to see the ICD used in cases considered under the Police Pensions Regulations 1987 and the Police (Injury Benefit) Regulations 2006. This is because Home Office guidance on medical appeals under these regulations (now archived and being updated) refers to how ‘The police authority should require the SMP [selected medical practitioner] to describe wherever possible any disease or medical condition causing disablement by reference to internationally authoritative guides available to doctors such as ICD 10 […] and DSM IV’ (Home Office 2007). However, the words ‘wherever possible’ and ‘such as’ should not be overlooked.

### Gender issue cases

In many cases where experts rely on, or refer to, both the DSM and ICD, the courts have been uncertain as to their relative status. This happened in *JR111, Re Application for Judicial Review* [2021], where the parties were unable to provide the court with much assistance as to which of the two taxonomies was in more prevalent use in clinical practice in the UK or when and how a psychiatrist or psychologist would use one rather than the other, although it was recognised that previous UK authorities in the particular field of gender issues seemed to place greater emphasis on the ICD classification.

The issue was whether, in order to secure a gender recognition certificate under the Gender Recognition Act 2004, a transgender person was still required to show, among other things, that they had ‘gender dysphoria’, defined by the Act as ‘the disorder variously referred to as gender dysphoria, gender identity disorder and transsexualism’ (para. 25). The claimant objected to this on both principled and practical grounds. It was submitted on her behalf that the requirement for a diagnosis of a gender identity disorder ‘irrationally requires transgender people to say that their understanding of their gender is caused by a mental disorder rather than a normal function of human variation’. It was also averred that the ‘pathologisation’ of transgender people was now out of line with international best practice, including by reference to ICD-11. This was because, although DSM-5 emphasises that incongruence is the ‘core component’ of a diagnosis of gender dysphoria, there remains an emphasis on clinically significant distress or impairment that does not find similar expression in the ICD’s description of gender incongruence. The applicant succeeded because the court found that, notwithstanding the uncertainty as to the relative status of the ICD and DSM, the requirement to provide a specific diagnosis which was defined as a ‘disorder’ in order to secure a gender recognition certificate failed to strike a fair balance between the interests of the applicant and those of the community generally.

### Criminal cases

One of the most critical considerations of the ICD and DSM in any proceedings has been that of Lord Justice Hughes in *R v Dowds* [2012], where, quoting from the introductions to the ICD and DSM, including the cautionary section in the latter, he applied the concept of the ‘imperfect fit’ between questions of ultimate concern to the law and the information contained in a clinical diagnosis to the case in point.

## Discussion

### Consensus – but what sort of consensus?

Neither the ICD nor DSM, as first developed, was intended to be used in legal proceedings. The ICD was developed for the use of a wide range of health professionals in various countries and the DSM for psychiatrists and psychologists in the USA, but neither was developed for psychiatrists and psychologists seeking to assist the courts. Although both are regarded as categorical systems, mental disorders are non-taxonomic. There is an artificiality to the discrete categories, within which many people with mental disorders cannot be placed on the application of the DSM’s algorithmic criteria or ICD’s prototypical matching to the detailed descriptions in its guidelines.

Although the DSM and ICD have evolved over 60 years, they have evolved little in response to the identification of any objective laboratory measure or biological marker for a mental disorder or research establishing how a particular disorder can be sufficiently distinguished from other similar disorders, in terms of epidemiology, response to treatment and prognosis, even though the specificity with which diagnoses direct treatment or predict outcome is a major rationale for any diagnostic system. Rather, they have evolved through the work of committees that achieve consensus, but committees reportedly influenced by the pharmaceutical industry, patient advocacy groups, psychiatrists who may be motivated by the acquisition of status, members with financial interests and what have been described as political horse-traders. Furthermore, scientific consensus is a distributed, emergent property of the scientific community as a whole:

‘It is not something that can be discovered or created by a particular group of scientists within an institutional committee. An individual scientist or group of scientists can attempt to summarize the state of scientific discourse and propose their own model, but this is necessarily imperfect, especially in discussing something as conceptually and theoretically broad and vague as mental disorder’ (Markon 2013).

The ICD and DSM may represent consensus but to suggest that it is scientific consensus is misleading.

It is unsurprising that in 1993 Gunn et al advised:

‘The doctor thus must attempt to determine the existence of any psychiatric disorder and its relation to the incident. The court is more concerned with the existence of disorder in itself, its attribution, and its consequences than with niceties of diagnosis and classification. Diagnostic terms should be used simply and conventionally, but it is unnecessary to follow slavishly definitions from textbooks and glossaries such as DSM III R or ICD-9’ (Gunn 1993: p. 102–3).

Nevertheless, for 30 years or more, courts and tribunals have increasingly relied on the ICD and DSM. They are used in order to decide whether a claimant or plaintiff in a personal injury case, particularly in Scotland, or in an employment case has a psychiatric injury or recognised/recognisable psychiatric illness. Their use may have become *de rigueur* in immigration and asylum cases, but there is no evidence of a widespread view that failure to use them is indicative of a lack of rigour, as happened in *DE (suicide, psychiatric treatment, J applied) Turkey* [2005].

### Struggles for litigants and struggles for experts

Perhaps because the ICD and DSM have become well-known in some jurisdictions, although it remains unnecessary to apply their criteria or descriptions slavishly, it may now be necessary in some jurisdictions to use them. In their book on litigating psychiatric injury claims, Marshall et al (2012) advocate the use of the ICD or DSM as ‘a claimant is likely to have an uphill struggle to achieve compensation without a psychiatric condition that will fit one, or more, of the diagnostic descriptions’ (p. 129).

Given the limitations of the ICD and DSM, it is inadvisable to struggle to apply a DSM or ICD diagnosis to the subject of a medico-legal psychiatric assessment, forcing a square peg into a round hole and risking the weaknesses of the classification and process being used to undermine the diagnosis and reduce the weight of the psychiatric evidence. The court may be better assisted by explaining why, even though unable to apply a DSM or ICD diagnostic label to the individual, they have what a responsible body of psychiatrists or psychologists would recognise as an injury, illness or disorder and justify this by reference to the range and severity of the psychopathology and evidence of impairment of functioning.

### Diagnostic label or clinical formulation?

Although designed to avoid having to use in communication what Tyrer (2014) has described as ‘a lengthy description of clinical problems’, this may be just what the court needs or at least what ICD-11 terms ‘a diagnostic formulation appropriate for an individual patient, considering the patient’s individual, social and cultural context’. It may be worth pointing out that, as stated in the introduction to ICD-11 (WHO 2024), its focus (and that of the DSM) is on the classification of disorders and not the assessment and treatment of people, who are frequently characterised by multiple disorders and diverse needs. They are classifications of disorders and not classifications of people.

What the courts and clinicians share is the need sufficiently to understand, for their differing purposes, the nature, severity, effects, and sometimes the causes, treatment and prognosis, of a person’s mental condition. The process of clinical formulation (Baird 2017) should be the starting point. From a clinical formulation concise statements can be made, as answers to questions or opinions on issues, in relation to such matters as the nature, severity and effect of the person’s condition, causation, treatment and prognosis. Just as clinical formulation helps clinicians understand their patient’s condition, its adaptation in medico-legal reporting assists the court in better understanding the person whose mental, behavioural or neurotypical condition is in issue. Diagnostic labelling, whether using the DSM, ICD, some other classification or even no classification at all, is only a part of the process.

Inappropriate use or reliance on authoritative classifications of disorders must not impede or risk preventing the delivery of justice to people who choose, or are forced, to go to law.

### Conclusion

Courts and tribunals in the British Isles often receive psychiatric evidence that relies on the DSM and ICD classifications and some expect reference to one or the other or even both. However, some courts are unaware of their limitations and the risks of using them to answer what DSM-5 calls ‘questions of ultimate concern to the law’. Experts who rely on them without also referring to their limitations risk exposure when their evidence is tested in court and, worse still, risk misleading the court and impeding or even preventing the delivery of justice.

MCQs

Select the single best option for each question stem

1. The ICD and DSM classifications:
  - rely on mental disorders being non-taxonomic
  - are of particular value as they can be used to make diagnoses in the majority of patients
  - are based on scientific consensus
  - have as their focus the assessment of people who may have mental disorders
  - are better regarded as nomenclatures than classifications.
2. About the ICD:
  - the ICD can be traced back to Brigadier General William Menninger, MD, who served as Chief of the US Army Medical Corps’ Psychiatric Division
  - it is not used in the USA
  - it represents international consensus as to the detailed descriptions of conditions that are used for a process mainly of algorithmic diagnosis
  - each successive edition aims to provide comprehensive statements about the current knowledge of the disorders
  - since ICD-6 the public health focus of the ICD has progressively diminished.
3. About the DSM:
  - it is a comprehensive classification of mental, behavioural and neurotypical disorders for use by a wide range of health professionals in countries of very varied sizes, cultures and resources
  - when DSM-5 categories, criteria and textual descriptions are employed for forensic purposes there is a risk that diagnostic information will be misused or misunderstood
  - it relies on there being zones of rarity between different disorders
  - the first version originated in the first International Statistical Congress in Brussels in 1853
  - in each successive edition changes can be made only on the basis of research findings.
4. Which of the following represents guidance on the use of ICD and DSM?
  - statements about the duration of symptoms in ICD are intended as strict requirements
  - in most situations, DSM or ICD diagnoses will be sufficient to establish the existence for legal purposes of a mental disorder, mental impairment or psychiatric injury
  - the use of DSM-5 should be informed by an awareness of the risks and limitations of its use in forensic settings
  - both the ICD and DSM are written so as to avoid the need to exercise clinical judgement in choosing diagnoses
  - an ICD or DSM diagnosis obviates the need for a clinical formulation.
5. In courts and tribunals in the British Isles:
  - for a successful personal injuries claim based on post-traumatic stress disorder in England and Wales the Judicial College Guidelines require a DSM diagnosis
  - in the Employment Tribunal and in police pension cases, where mental disorder is an issue, the only classifications recognised are the ICD and DSM
  - it is accepted in the Health, Education and Social Care Chamber (Mental Health) that decisions of the First-tier Tribunal will depend on which classification is used
  - the advantage of the DSM over the ICD is that it is designed to assist in answering questions of ultimate concern to the law
  - it is common practice in personal injury pleadings in Scotland to aver that a pursuer was or is suffering from a specified condition that is recognised in the DSM or ICD.

MCQ answers

1. e
2. a
3. b
4. c
5. e

## Data Availability

Data availability is not applicable to this article as no new data were created or analysed in this study.
